# Vocational rehabilitation to enhance return to work after trauma (ROWTATE): protocol for a non-randomised single-arm mixed-methods feasibility study

**DOI:** 10.1186/s40814-021-00769-4

**Published:** 2021-01-20

**Authors:** Denise Kendrick, Roshan das Nair, Blerina Kellezi, Richard Morriss, Jade Kettlewell, Jain Holmes, Stephen Timmons, Kay Bridger, Priya Patel, Adam Brooks, Karen Hoffman, Kathryn Radford, On behalf of the ROWTATE study team

**Affiliations:** 1 School of Medicine, University Park, Floor 13, Tower Building, Nottingham, NG7 2RD UK; 2 School of Medicine, Institute of Mental Health, Jubilee Campus, Wollaton Road, Nottingham, NG8 1BB UK; 3 Division of Psychology, School of Social Sciences, Nottingham Trent University, Burton Street, Nottingham, NG1 4BU UK; 4 Centre for Health Innovation, Leadership and Learning, Nottingham University Business School, Jubilee Campus, Wollaton Road, Nottingham, NG8 1BB UK; 5 School of Medicine, Medical School, Queen’s Medical Centre, Nottingham, NG7 2UH UK; 6 Centre for Trauma Sciences, Blizard Institute, Barts and The London School of Medicine and Dentistry, Queen Mary University of London, 4 Newark St, London, E1 2AT UK; 7 Nottingham University Hospitals NHS Trust, Queen’s Medical Centre, Derby Road, Nottingham, NG7 2UH UK

**Keywords:** Vocational rehabilitation, Work, Occupation, Trauma, Injury, Psychological outcomes

## Abstract

**Background:**

Traumatic injuries are common amongst working-age adults. Survivors often experience physical and psychological problems, reduced quality of life and difficulty returning to work. Vocational rehabilitation improves work outcomes for a range of conditions but evidence of effectiveness for those with traumatic injuries is lacking. This study assesses feasibility of delivering a vocational rehabilitation intervention to enhance return to work and improve quality of life and wellbeing in people with at least moderate trauma to inform design of a definitive randomised controlled trial (RCT).

**Methods:**

Non-randomised, single-arm, multi-centre mixed-methods feasibility study with nested case studies and qualitative study. The case studies comprise interviews, observations of clinical contacts and review of clinical records. The qualitative study comprises interviews and/or focus groups. Participants will be recruited from two UK major trauma centres. Participants will comprise 40 patients aged 16–69 with an injury severity score of > 8 who will receive the intervention and complete questionnaires. Interviews will be conducted with 10 patients and their occupational therapists (OTs), clinical psychologists (CPs), employers and commissioners of rehabilitation services. Fidelity will be assessed in up to six patients by observations of OT and CP—patient contacts, review of patient records and intervention case report forms. OT and CP training will be evaluated using questionnaires and competence to deliver the intervention assessed using a team objective structured clinical examination and written task. Patients participating in and those declining participation in the study will be invited to take part in interviews/focus groups to explore barriers and facilitators to recruitment and retention. Outcomes include recruitment and retention rates, intervention fidelity, OT and CP competence to deliver the intervention, experiences of delivering or receiving the intervention and factors likely to influence definitive trial delivery.

**Discussion:**

Effective vocational rehabilitation interventions to enhance return to work amongst trauma patients are urgently needed because return to work is often delayed, with detrimental effects on health, financial stability, healthcare resource use and wider society. This protocol describes a feasibility study delivering a complex intervention to enhance return to work in those with at least moderate trauma.

**Trial registration:**

ISRCTN: 74668529. Prospectively registered on 23 January 20

## Background

Injuries adversely affect working-age adults; they are the leading cause of death amongst 15–29 year olds and the second leading cause of death amongst 30–49 year olds worldwide [1]. In England and Wales in 2016, they were the third leading cause of death between 15 and 64 years of age (10,700 deaths) [2]. In 2015–2016, they resulted in more than 700,000 hospital admissions in England amongst those aged 16 and 69 years [3].

Significant concerns about survival rate post-injury [4, 5] resulted in the establishment of 22 major trauma centres (MTCs) for adults across the UK in the last decade. Patients with at least moderate trauma (injury severity score > 8) are now transported directly to MTCs. Survival rates have since improved [6], but many survivors experience physical and psychological problems [7, 8], reduced quality of life (QoL) [9] and difficulty returning to work [10, 11], with psychological and occupational needs frequently being unmet [12]. The detrimental effects of being out of work on health, healthcare resource use and wider society are well documented [13, 14]. We recently found that one third of patients admitted to hospital with an injury had not returned to work 1 year later, with many suffering significant physical and psychological problems which reduced the likelihood of returning to work [15, 16].

Moderate or severe traumatic injury often involves multiple physical injuries, affecting several body regions, frequently with psychological and/or cognitive problems impacting on work ability. These patients can face specific challenges in returning to work such as anxiety, depression and PTSD resulting from the injury [15, 17–20], pain [21], cognition problems [22], fatigue [23] and other hidden disabilities (e.g. urological problems following pelvic fracture) [24]. One year post injury, around one third of trauma centre patients report depression and PTSD [25], 22% of patients with musculoskeletal injuries report moderate or severe pain [26], just over one third of patients with traumatic brain injury report fatigue [23], and around half of patients with mild traumatic brain injury report cognitive problems [27].

Vocational rehabilitation can be defined as ‘a multi-professional approach that is provided to individuals of working age with health-related impairments, limitations, or restrictions with work functioning and whose primary aim is to optimize work participation’ [28]. Systematic reviews demonstrate that vocational rehabilitation improves employment outcomes across a range of conditions (for example, brain or spinal cord injury, back pain, mental health problems [29–33]). However, current evidence on vocational rehabilitation addresses single conditions, conditions affecting single body regions or psychological or physical problems, not both [29–34]. Systematic reviews suggest that the mechanisms for success in vocational rehabilitation are early intervention, coordinated multidisciplinary approaches, the ability to work across health and employment sectors and employer engagement [21, 35, 36]. However, none of the primary studies within these reviews examined vocational rehabilitation for patients with a diverse range of traumatic injuries.

UK National Health Service (NHS) patients admitted to MTCs are currently unlikely to receive optimal support to return to work, with uncertainty whether adequate rehabilitation services for these patients exist [37], inequitable access to rehabilitation services [38] and sub-optimal use of rehabilitation prescriptions [39].

We hypothesise that enhancing return to work in patients with at least moderately severe trauma will improve physical and psychological health, quality of life and financial stability and reduce healthcare costs. However, vocational rehabilitation services cannot be commissioned without good underpinning evidence. This study therefore assesses the feasibility of delivering a vocational rehabilitation intervention to enhance return to work and improve quality of life and wellbeing in people with at least moderate trauma to inform design of a definitive randomised controlled trial (RCT). For the purposes of this study, when we refer to ‘work’, this includes paid work, voluntary work and full-time education. This feasibility study is part of a 6-year programme of research (Multicentre Research Programme to Enhance Return to Work after Trauma (ROWTATE): https://www.rowtate.org.uk/) which includes intervention development, a feasibility study, a definitive RCT with nested economic and qualitative studies and an implementation study.

## Methods

### Objectives

Objectives of the feasibility study are as follows:

1. Assess recruitment and follow-up rates, data collection tools, processes and data completeness
2. Deliver the ROWTATE intervention and evaluate and optimise intervention usage and acceptability
3. Evaluate ways to assess and minimise contamination in a future trial
4. Assess intervention fidelity
5. Evaluate occupational therapist (OT) and clinical psychologist (CP) training to deliver the intervention
6. Evaluate importance and acceptability of outcome measures
7. Identify factors that may affect the running of the definitive trial, including barriers and facilitators to recruitment and retention

### Study design

**Fig. 1:**
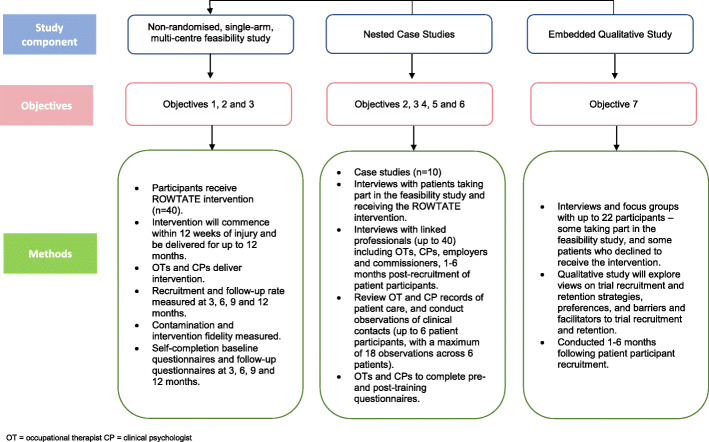
Study design and components

Non-randomised, single arm, multi-centre, mixed-methods feasibility study with nested case studies and qualitative study. The case studies comprise interviews, observations of clinical contacts and review of clinical records. The qualitative study comprises interviews and/or focus groups (Fig. 1). A non-randomised design has been chosen because the subsequent RCT will include an internal pilot. This will allow assessment of recruitment and retention rates against pre-specified progression criteria, and data collected during the internal pilot will contribute to the trial analysis. For this reason, internal pilots are potentially more cost-effective than running a randomised feasibility study followed by a full trial [40].

### Participating centres

Participants will be recruited from East Midlands MTC, Nottingham University Hospitals NHS Trust, and the MTC, Royal London Hospital, Bart’s Health NHS Trust. The two participating sites have diverse study populations in terms of age, ethnic group, deprivation and types of injury.

### Study population

Participants for this study are patients, OTs, CPs, employers and commissioners.

#### Study population for patients taking part in the feasibility study and receiving the ROWTATE intervention

Adult patients admitted to a participating MTC will be recruited up to 12 weeks post injury to allow inclusion of patients unable to consent earlier in their recovery, for example, those in intensive care units or those with cognitive impairment.

Inclusion criteria are as follows:

- Aged 16–69 years (lower age limit chosen as this is minimum age for leaving full time education and upper age limit chosen to reflect increased state pension age and changes to working patterns in the UK)
- Injury Severity Score > 8 as assessed on admission
- Employed, self-employed, in full-time education or voluntary work at the time of injury
- No plans to retire within the next year
- Not participating in other vocational rehabilitation trials
- Sufficient proficiency in English to contribute to the data collection required for research or willing to use approved interpreting service for data collection
- Not returned to work/voluntary work/education for ≥ 80% of pre-injury hours
- Able to give informed consent
- Resident in the catchment area of one of the two participating MTCs

Exclusion criteria are as follows:

- No fixed address where participants can be contacted for follow-up

#### Study population for case studies

Patients participating in the feasibility study and receiving the intervention and their linked OTs, CPs, employers and commissioners

#### Study population for qualitative study

Feasibility study patient participants who express interest in the qualitative study, plus patients who declined to participate in the feasibility study

### Participant identification and consent procedures

A list of potentially eligible patients will be produced by MTC staff, and patients will be approached by a member of the usual care team. Those agreeing to discuss the study with a member of the clinical or research team will be visited on the ward or at home to discuss the study and obtain written informed consent. Those discharged to other in-patient facilities prior to being approached in the MTC will be approached by the usual care team in those facilities; those discharged home will be approached by phone or letter. Those expressing interest will be recruited by a member of the usual care or research team. Patient recruitment will take place between March 2020 and June 2021.

Patient participants will be asked to express interest in taking part in the nested case studies and qualitative study. The qualitative study will include feasibility study patient participants who expressed interest in the qualitative study, plus patients who declined to participate in the feasibility study. For both the case studies and the qualitative study, patients expressing interest will be purposively sampled to represent a range of ages, genders, ethnicities, clinical characteristics (type of injury, severity of injury, mechanism of injury, place of injury), employment type (employed/self-employed/voluntary work/full-time education), employer size (micro 0–9 employees, small 10–49 employees, medium 50–249 employees and large ≥ 250 employees) [41] and rurality (distance from the MTC). For both studies, patients will be approached to take part until the required sample size is reached.

For the case studies, written informed consent will be obtained from patients to participate in interviews, observations of clinical contacts and to access clinical records. All OTs and CPs caring for study patients, patients’ employers, as well as the commissioners commissioning care for the patients’ geographical area, will be approached until the required sample size is reached. OTs, CPs, employers and commissioners will provide written informed consent for face-face interviews or verbal (recorded) consent for telephone interviews. OTs and CPs will provide written informed consent for observations of clinical contacts. Patients participating in interviews will receive a £20 gift voucher to thank them for their time. Case studies will take place up to 12 months from the date of patient recruitment.

For the qualitative study, written informed consent will be obtained for face-face interviews or focus groups or verbal (recorded) consent for telephone interviews. Patients participating in interviews or focus groups will receive a £20 gift voucher to thank them for their time. The interviews or focus groups for the qualitative study will take place up to 6 months from the date of patient recruitment.

### Data collection

#### Data to address objective 1

Data will be collected to describe baseline characteristics of the study sample and of a range of outcome measures including work outcomes, health status measures and health and social care resource use data that may be suitable for use in the subsequent randomised controlled trial and its economic evaluation.

The following baseline data will be collected from patient participants at recruitment to the study using self-completed questionnaires:

- Demographic details
- Injury details
- Employment or education details (before injury and since injury)
- Health and wellbeing since injury: quality of life (EQ-5D-5L) [42]; Trauma Outcome Profile (pain, physical and mental function, social interaction, body image, satisfaction) [43]; depression, anxiety, (Hospital Anxiety and Depression Scale (HADS [44]) and post-traumatic stress (Impact of Event Scale (IES) [45]); recovery expectations [46]
- Work since injury: Return to Work Self Efficacy Scale [47], single-item question from the Work Ability Index [48] and the Work Limitations Questionnaire [49], bespoke questions on support received to return to work
- Financial impact: 5 items from the Chronic Stress scale [50] and 2 items from the Family Transitions Project [51]
- Health and social care resource use since injury using bespoke questions designed to collect health economic data including use of secondary care, primary care, other community-based services, social care and informal care

Follow-up data will be collected from patient participants 3, 6, 9 and 12 months after recruitment by self-completion questionnaire and will comprise the same data collected at baseline excluding demographic and injury details and employment or education details before injury. In addition, following recommendations from our patient and public involvement group, the follow-up questionnaire will include the Purpose in Life scale [52]. To thank them for their time, participants will receive a £10 gift voucher for each completed questionnaire. Two reminders (paper, phone, text, online) will be used to increase response rates. Researchers will assist participants requiring help to complete questionnaires.

#### Data to address objective 2

The number, content, duration and frequency of contacts between OTs or CPs and patient participants and their employers will be recorded by OTs and CPs on intervention delivery case report forms (CRFs). Case study interviews with patients, OTs, CPs, employers and commissioners will explore factors which may influence delivery of the intervention and how it could be improved as well as acceptability of the intervention (see Table 1 for interview topics).

**Table 1:** Interview topics for case study interviews

Interview topics	Interviewees			
	Patient	OTs and CPs	Employer	Commissioner
Experiences (positive and negative) of ROWTATE intervention	x	x	x	
Experiences and decision-making around returning to work (or not) post-injury (including aspects such as discrimination, transport issues, work-life balance)	x			
Factors facilitating or hindering engagement with the intervention or return to work	x	x	x	x
Mechanisms considered important in determining key outcomes	x	x	x	
Acceptability of the trial procedures	x	x		
Importance and acceptability of outcome measures	x	x	x	x
Perceived appropriateness of timing of return to work	x	x	x	
Readiness to deliver the ROWTATE intervention following training		x		
Potential improvements to training		x		
Potential contamination issues		x		
Information on the employing organisation and its policies and practices to support return to work			x	
Potential improvements to the ROWTATE intervention		x	x	
Policy drivers for commissioning vocational rehabilitation				x
Service quality factors and contracting issues of importance				x

#### Data to address objective 3

Potential sources of contamination that may occur in a future trial and possible ways of preventing or minimising them will be identified and recorded by OTs and CPs during therapist training, intervention delivery or mentoring sessions. They will also be identified through case study interviews with OTs and CPs (described in “Data to address objective 2” section above). Such issues could include, but are not limited to, OTs or CPs not taking part in the feasibility study accessing ROWTATE training materials, treatment records or other intervention knowledge or exposure to the intervention, for example, being supervised by OTs or CPs delivering the ROWTATE intervention; ROWTATE intervention OTs and CPs delivering care to patients not taking part in the feasibility study; plans for service development or new initiatives to meet MTC patients unmet work needs or any other potential contamination issues identified by OTs, CPs or their mentors.

#### Data to address objective 4

Data collected to assess intervention fidelity will include intervention delivery CRFs detailing intervention content for each patient participant contact, OT and CP mentoring records, video or audio-recorded observations of OT and CP clinical contacts with patient participants and review of clinical records of intervention contacts. These data will be used to complete a fidelity template based on the Conceptual Framework for Implementation Fidelity [53] for each patient participant. Communication skills will be assessed by the observer completing the Maastricht MAAS—Global Rating List for Consultation Skills [54] focussing on communication skills for phases of the encounter and general communication skills.

#### Data to address objective 5

The following baseline data will be collected from OTs and CPs immediately prior to intervention training by self-completion questionnaire:

- Demographic details
- Qualifications
- Work experience

Follow-up data will be collected from OTs and CPs immediately after training by self-completion questionnaire and will comprise 4 items from the Evidence-Based Practice Confidence (EPIC) Scale [55], the Evidence-Based Practice Attitude Scale (EB-PAS) [56] and bespoke questions on confidence in delivering the ROWTATE intervention. Qualitative data on readiness to deliver the ROWTATE intervention will be collected from case study interviews with OTs and CPs (described in “Data to address objective 2” section above). Quantitative data on OT and CP competency to deliver the ROWTATE intervention will be collected by a team objective structured clinical examination (TOSCE) at the end of the training session, together with an individual and a team written task. The TOSCE is situated around a case study using a professional actor for the OTs and CPs to interact with. The individual written task is a letter to the employer, and the team written task is a clinical summary and formulation for the proposed intervention based on the case study. Competency results will be provided to the OTs and CPs by the training team and used by the mentors to support intervention delivery.

#### Data to address objective 6

Case study interviews (described in “Data to address objective 2” above) will be used to evaluate the importance and acceptability of outcome measures.

#### Data to address objective 7

Case study interviews (described in “Data to address objective 2” above) will be used to identify factors that may affect the running of the definitive trial. Focus groups or interviews in the qualitative study with feasibility study patient participants, plus patients who declined to participate in the feasibility study, will explore views on trial recruitment and retention strategies, preferences, and barriers and facilitators to trial recruitment and retention.

### Intervention

ROWTATE adopts a coordinated multidisciplinary and multi-stakeholder approach to solving the problems presented by the person wanting to return to work after injury [21, 29, 31, 36]. It is an adaptive form of rehabilitation targeted at facilitating participation in meaningful occupation. It requires the OT and CP to adopt different evidence-based treatment approaches and to intervene at different levels, for example, at the remedial level, remediating injury-related problems such as mobility and low mood but also adaptation, teaching patients to manage pain, anxiety or fatigue and adapt to life and work with more permanent disability, e.g. spinal injury or disfigurement.

Different ‘treatment’ theories underpin the individual approaches, for example, in Cognitive Behavioural Therapy (CBT), the ‘cognitive model’ is used as a framework to understand mental distress. CBT helps patients understand how they think and behave and equips them with the tools to change their maladaptive cognitive and behavioural patterns.

In ROWTATE, these treatment theories [57] sit within a broader Enablement Theory—The International Classification of Function (ICF) [58]—a bio-psychosocial framework that is useful for thinking about vocational rehabilitation in terms of the relationship between the person, their ability to engage in work activities and meaningful life roles and the context in which this takes place from a personal (confidence, choice and experience, attitudes and expectations) and environmental (workplace, co-workers, job type, employer attitudes, family influence) perspective. It therefore allows us to consider the dynamic interaction between the injury and contextual influences on return to work.

The ICF recognises the importance of the social environment such as employment type or enterprise size as a determinant of work outcomes, for example, in supporting a person with a spinal injury to return to work by adapting the workplace and the job to accommodate the wheelchair (disability); at a social level, by recognising the importance of the relationship with the line manager, the employers attitudes to disability, and having adequate family support and at the psychological level by addressing the patient’s pre-morbid coping styles, confidence, beliefs and motivation. These factors may be more important determinants of work outcome than the nature of the injury itself [21, 30, 35, 59].

The intervention has been informed by similar interventions previously developed by our team for patients with traumatic brain injury and stroke [60, 61]. It has been developed by a multi-disciplinary team comprising OTs, clinical and neuropsychologists, a psychiatrist, vocational rehabilitation experts, a general practitioner, specialists in rehabilitation and trauma medicine, social scientists and patient representatives. We used the person-based approach [62] to intervention development informed by interviews, focus groups, co-production workshops and talking through care pathways across five MTCs which will take part in the definitive RCT (Queen’s Medical Centre, Nottingham; Royal London Hospital; Addenbrooke’s Hospital, Cambridge, Leeds General infirmary; Southmead Hospital, Bristol). Participants included OTs, clinical and neuropsychologists, psychiatrists, a psychotherapist, physiotherapists, vocational rehabilitation experts, general practitioners, specialists in rehabilitation and trauma medicine, trauma network directors, solicitors, case managers, disability employment advisors, trauma survivors and carers. Drawing on this qualitative data, we also used soft systems methodology [63] to understand the context for implementing the intervention, including service gaps (by identifying how and where work issues are currently addressed for trauma survivors in the five MTCs) and local unmet need (what vocational rehabilitation services do they currently receive?). An iterative process was used to develop and refine the logic model for the intervention. Further details about development of the intervention will be published separately.

The ROWTATE intervention will be provided to patients meeting study eligibility criteria who give informed consent. Recruitment will continue until 40 participants have been recruited. The intervention will commence within 12 weeks of injury and be tailored in content, duration and frequency according to individual need and OT and CP clinical judgement over a 12-month period. An OT will work in a case coordinator role with a wider team of healthcare professionals, employers, family members and other agencies (e.g. solicitors, insurance agencies) to:

- Assess the impact of the injury on the patient participant and their family and on their role as a worker
- Educate patient participants, employers and families about the effects of the injury and its impact on work and find acceptable strategies to lessen the impact
- Continually monitor and assess the patient participant’s post-injury life and work goals
- Prepare patient participants for work by establishing structured routines with gradually increased activity levels and opportunity to practice work skills
- Liaise with employers, employment advisers, solicitors and the healthcare team to advise about the effects of the injury and to negotiate, plan and monitor a phased return to work
- Screen for mental health problems and refer to a clinical psychologist for assessment and provision of evidence-based approaches for managing trauma-related mental health issues as needed. Complex cases will be managed following a formulation agreed jointly by the OT and CP.

OTs and CPs will be supported to deliver the ROWTATE intervention by an OT or CP experienced in providing rehabilitation for those with serious injuries and in vocational rehabilitation through monthly telephone mentoring.

### Outcomes

The following feasibility study outcomes will be measured:

- Recruitment rate and follow-up rate at 3, 6, 9 and 12 months
- Patient participant preferences for paper/online/telephone versions of the outcome data collection tools, number and type of reminders required and data completeness. Measured using self-completion questionnaires at recruitment and follow-up.
- Patient participant, OT, CP, employer and commissioner experiences of the ROWTATE intervention and of returning to work (or not); factors facilitating or hindering engagement with the intervention or return to work; perceived appropriateness of timing of return to work; mechanisms considered important in determining key outcomes; importance and acceptability of outcome measures; acceptability of trial procedures; information on the employing organisation and its policies and practices to support return to work; potential improvements to the ROWTATE intervention; policy drivers for commissioning vocational rehabilitation, and service quality factors and contracting issues of importance. Measured using semi-structured interviews 1–12 months post patient participant recruitment.
- Identification of potential contamination issues. Measured by completion of contamination and mentoring records throughout the intervention period.
- Fidelity of and contextual and process issues related to intervention delivery. Measured by completion of intervention delivery CRFs, observations of OT and CP contacts with patients, assessment of communication skills, review of clinical records, completion of mentoring records 0–12 months post patient participant recruitment.
- OT and CP readiness to deliver the ROWTATE intervention following training and potential improvements to training. Measured using self-completion questionnaires (Evidence-Based Practice Confidence Scale, Evidence-Based Practice Attitude Scale and bespoke questions on confidence to deliver intervention components) before and immediately after training. Readiness to deliver the ROWTATE intervention will be measured using semi-structured interviews 1–6 months post patient participant recruitment. Competence to deliver the ROWTATE intervention will be measured by a TOSCE, plus an individual task (letter to an employer) and team-completed task (completion of clinical summary) immediately after training.
- Patient participants’ views on trial recruitment and retention strategies, preferences, and barriers and facilitators to trial recruitment and retention. Measured by focus groups and/or interviews 1–6 months after patient participant recruitment.

### Sample size

We will recruit 40 patient participants to take part in the feasibility study and receive the intervention. Sample sizes of 40–50 participants in total have been recommended for feasibility studies [64] and we consider 40 participants will enable us to address objectives 1 to 3.

To address objectives 2, 3, 4 and 6, we will conduct 10 case studies, including interviews with patients taking part in the feasibility study and receiving the intervention (*n* = 10) and their linked professionals (up to 40 in total), review OT and CP records of their care, and conduct observations of clinical contacts (up to 6 patient participants, with a maximum of 18 observations across the 6 patient participants). To address objective 5, all OTs and CPs delivering the intervention will complete pre and post training questionnaires, the TOSCE and the individual- and team-completed tasks.

To address objective 7, we will conduct focus groups and/or interviews with up to 22 patients who did and did not participate in the feasibility study. We anticipate this will allow ‘theoretical sufficiency’ [65] as opposed to data saturation, but further interviews will be conducted if we do not achieve theoretical sufficiency. Using ‘conceptual depth criteria’, we will look for range, complexity, subtlety, resonance and validity in the data [66].

### Adverse events

This is a non-pharmacological intervention study in a population likely to have repeated contacts with the healthcare system including hospital admissions related to their original injury. Serious and unexpected adverse events in relation to participating in this study are likely to be rare events. Consequently, the risks of patient harm arising from a low-risk intervention must be weighed against collecting large amounts of data on unrelated events. For the purposes of this study we define adverse events as hospital admissions and workplace accidents requiring medical attention. We will also collect data on ‘potential harms’ including workplace accidents not requiring medical attention and ‘near miss’ accidents. Data on adverse events and potential harms will be collected prospectively using specific patient participant completed reports and by reporting events on follow-up questionnaires. Adverse events and potential harms will also be reported by OTs, CPs and researchers who have contact with patient participants. Adverse events and potential harms will be reviewed and monitored by a subgroup of the programme steering committee.

### Data management

All study data will be handled confidentially. Participants will be assigned a unique identity code number for use on CRFs, other study documents and electronic databases. Paper records will be stored in locked cabinets and electronic records on password-protected databases. Identifiable data will be stored separately from non-identifiable data.

Quantitative data will be entered into a bespoke access database by members of the research team. Data will be checked by a member of the research team who did not complete the original data entry. Qualitative data will be anonymised and managed using the NVivo software (Version 12).

### Statistical analysis

Recruitment and retention in the feasibility study will be shown using the flow chart from the CONSORT 2010 statement: extension to randomised pilot and feasibility trials [67], adapted to include only the intervention arm. Reasons for ineligibility and for non-participation will be reported.

The recruitment rate and retention rate (number providing data on return to work on follow-up questionnaires at 3, 6, 9 and 12 months) and 95% confidence intervals will be calculated. The numbers and reasons for study withdrawals will be reported, as will patient participant preferences for questionnaire administration methods and reminders required.

Data completeness for outcome measures will be reported as frequencies and percentages for single item questions and the mean number of items (and standard deviation (SD) or median and interquartile range as appropriate) completed for scale measurements.

Frequencies and percentages will be reported for the number and content of intervention contacts, potential contamination issues identified and for demographic details, qualifications and work experience for OTs and CPs. We will sum item-level responses on the 4 EPIC questions to create a mean summary score [55]. EP-BAS total scale and sub-scale scores will be reported using means and SDs [56]. The mean (and SD) confidence in delivering the ROWTATE intervention for each item and across all items will be reported. Means and SDs for sub-scale scores for the Maastricht MAAS—Global Rating List for Consultation Skills [54] will be reported.

Using an observational checklist at the time of the TOSCE, predetermined tasks for each therapist, based on communicating the case history, discussing the clinical case, identifying pertinent questions and generating a clinical formulation will be independently rated by two researchers and assessed for agreement. Each therapist is identified by a letter, e.g. A, B or C on the observational checklist. Where a task is led by one therapist, the independent raters use the checklist to mark this lead individual as well as the cooperative interaction of the remaining two therapists on whether the pre-determined individual behaviour is not attempted, is attempted but not appropriate or attempted and appropriate. For example, task one requires the lead therapist ‘A’ to communicate the baseline questionnaire data related to the case study and the other two therapists ‘B’ and ‘C’ should interact and ask questions, clarify points to demonstrate a team approach. Findings from the TOSCE will be reported elsewhere. A written clinical formulation produced by the clinical team (two OTs and a CP from each site) and letters to the employer produced by each team member will be assessed against a model answer. Overall scores for each therapist, based on their knowledge of the intervention process (40%), clinical reasoning (50%) and written communication (10%) will be mapped to a rubric identifying the therapists as highly competent (≥ 70%), competent (50–69%) or needing additional support (≤ 49%).

Quantitative data will be analysed using SPSS v25.

### Qualitative analysis

Interviews and focus groups will be transcribed verbatim and analysed using framework analysis. As well as reporting fidelity assessment quantitatively, qualitative data from observations, review of clinical records, mentoring records and interviews will be used to identify those factors that moderate fidelity. Framework analysis will also be used to analyse this data, based on the conceptual framework for implementation fidelity [53] to identify barriers and facilitators associated with intervention delivery.

### Feasibility assessment criteria

The following criteria will be used to assess fidelity:

- ≥ 80% of required sample size is recruited, or where 50–79% of required sample size is recruited, strategies to increase recruitment are identified.
- < 40% of participants are lost to follow-up at 12 months and strategies to reduce to between 20 and 30% are identified.
- ≥ 90% of participants commence the intervention, or if < 90%, strategies to increase this are identified.
- < 30% participants withdraw from the intervention, or if ≥ 30%, strategies to reduce this are identified.
- Therapists adhere to the ROWTATE intervention process in 70% of cases in the case studies and where < 70% moderating factors can be explained.
- Participants engage in 70% of prescribed intervention sessions and where < 70% strategies to increase this can be identified.
- ≥ 67% of OTs and CPs are judged to be competent to deliver the intervention or where < 67% strategies to increase this are identified.

### Governance

A steering committee with an independent chair, independent statistician, independent expert clinician and independent PPI representative will provide oversight of the feasibility study. The study sponsor is Nottingham University Hospitals NHS Trust. The programme grant co-applicants shall establish an information governance committee to govern access to the data following completion of the research programme.

### Patient and public involvement

The design of the feasibility study has been informed by our ROWTATE PPI Group of ten patient and public involvement group representatives with lived experience of a variety of traumatic injuries and subsequent rehabilitation journeys. They have contributed extensively to study design, development of patient-facing study documents, choice of patient outcome measures collected at baseline and follow-up and interview and/or focus group topic guides. Four group members have contributed to the development and delivery of the OT and CP training to deliver the ROWTATE intervention. During the feasibility study, PPI group members will continue to be closely involved in study management, collection and analysis of qualitative data, writing of plain English summaries of reports and dissemination of study results.

### Dissemination

Participants will be sent a summary of the study findings, and the summary will be made available on the ROWTATE study website. Study findings will be published in academic and practitioner journals, presented at conferences and made available to relevant patient groups via a range of dissemination channels. Authorship will be based on the International Committee of Medical Journal Editors Recommendations for the Conduct, Reporting, Editing, and Publication of Scholarly Work in Medical Journals [68].

## Discussion

Effective vocational rehabilitation interventions to enhance return to work amongst moderately and severely injured trauma patients are urgently needed because return to work is often delayed, with consequent detrimental effects on health, financial stability, healthcare resource use and wider society. Our protocol describes a feasibility study delivering a complex intervention to enhance return to work in those with at least moderate trauma. Assessing the feasibility and acceptability of complex interventions is important to determine key parameters for definitive trials and explore uncertainties around procedures, processes and outcomes likely to affect the conduct of a definitive trial [69]. This is particularly true given that our intervention is novel and there is little published work evaluating vocational rehabilitation interventions in our patient population.

The strengths of our study are that it builds on previous work developing and testing the feasibility of delivering a complex intervention to facilitate return to work in patients with acquired brain injury and stroke [60, 61]. It is being undertaken in two study centres with diverse patient populations, differing geographies and service configurations, allowing exploration of context-specific factors that may influence running of the definitive trial. We will evaluate OT and CP competence to provide the intervention using a TOSCE. This is particularly relevant to our intervention where OTs and CPs need to work closely together to provide coordinated vocational rehabilitation, and the TOSCE will allow us to assess inter-professional teamwork competencies [70]. There are a number of challenges we are likely to face. The study population is a heterogeneous group in terms of injury types, mechanisms and severity. This will increase the generalisability of our findings to the population of injured patients, but recruiting and retaining participants with some injuries may be more difficult than others. For example, recruiting and retaining patients with brain injuries can be difficult [71]. Data is being collected on a wide range of outcome measures, which may be burdensome for some participants. Consequently, the number of outcome measures used in the definitive trial may need to be reduced. We plan to explore barriers and enablers to recruitment and retention amongst those who did take part in our feasibility and received the intervention and those who did, but we recognise it may be difficult to recruit this latter group of patients.

The feasibility study protocol described in this paper forms part of a National Institute for Health Research Programme Grant for Applied Research. If feasibility is demonstrated, the definitive RCT will commence recruitment in 2021.

## Data Availability

Not applicable

## Abbreviations

CP: Clinical psychologist
CRF: Case report form
EB-PAS: Evidence-Based Practice Attitude Scale
EPIC: Evidence-Based Practice Confidence Scale
HADS: Hospital Anxiety and Depression Scale
IES: Impact of Event Scale
MTC: Major trauma centre
NHS: National Health Service
OT: Occupational therapist
PPI: Patient and public involvement
REC: Research ethics committee
RCT: Randomised controlled trial
SD: Standard deviation
SPSS: Statistical Package for the Social Sciences
TOSCE: Team objective structured clinical examination
